# Role of B-scan ultrasonography in the localization of intraocular foreign bodies in the anterior segment: a report of three cases

**DOI:** 10.1186/s12886-015-0076-1

**Published:** 2015-08-14

**Authors:** Kaijun Wang, Jun Liu, Min Chen

**Affiliations:** Eye Center, The 2nd Affiliated Hospital, Medical College of Zhejiang University, No. 88 Jiefang Road, Hangzhou, 310009 China; Zhejiang Provincial Key Laboratory of Ophthalmology, Hangzhou, China

**Keywords:** Ocular trauma, Intraocular foreign bodies, B-scan ultrasonography, Localization

## Abstract

**Background:**

The accurate localization of intraocular foreign bodies (IOFBs) is very important for the management of ocular trauma patients. B-scan ultrasonography is usually used to detect IOFBs in the posterior segment. Here, we report three cases with IOFBs in the anterior segment near the posterior lens capsule, which were accurately localized by B-scan ultrasonography under dynamic transversal scanning.

**Case presentation:**

All three patients had a history of ocular trauma, and their clinical symptoms were compatible with the persistence of IOFBs. It was difficult to get a direct visualization of IOFBs with slit-lamp biomicroscopy because of opacities of the cornea and traumatic cataract. A computed tomography scan detected IOFBs in the anterior segment, but could not determine the exact location. Ultrasound biomicroscopy was performed but failed to show any IOFBs owing to the limited depth of penetration. B-scan ultrasonography was further applied but also failed to show any intraocular foreign bodies using axial scanning, a routine procedure of B-scan ultrasonography examination. However, using dynamic transversal scanning of B-scan ultrasonography, the accurate location of IOFBs was eventually shown to be embedded in the posterior lens cortex in case 1, adjacent to the posterior lens capsule in case 2, and located in the anterior vitreous close to the posterior lens capsule in case 3. Different surgical procedures were designed according to localization by B-scan ultrasonography, and all IOFBs were successfully removed.

**Conclusion:**

B-scan ultrasonography is a simple and effective imaging modality in the localization of IOFBs in traumatic cataract. Transversal scanning is more suitable than axial scanning to detect IOFBs in the anterior segment near the posterior lens capsule.

## Background

Intraocular foreign bodies (IOFBs), with an incidence of about 18–41 % [1], are commonly encountered in cases of penetrating ocular trauma. However, the lens is not commonly involved, and the incidence of intralenticular foreign bodies is only 5–10 % [2, 3]. Accurate localization of IOFBs is essential to evaluate the severity of the ocular lesion and to determine further management. Computed tomography (CT) scanning, ultrasound biomicroscopy (UBM), and B-scan ultrasonography are widely used procedures in the assessment of IOFBs. CT is considered the first-line imaging methodology, and the most sensitive method for characterizing ocular trauma in patients with a suspected IOFB [4]. UBM can provide superior images of the anterior segment and has been a valuable tool for the localization of IOFBs in this region [5]. Here, we presented three cases with traumatic cataract and IOFBs in the anterior segment. Compared with CT and UBM, B-scan ultrasonography was used to identify the exact location of the IOFBs and to assess the status of the posterior lens capsule. To our knowledge, this is the first report describing the use of B-scan ultrasonography for accurate localization of IOFBs in the anterior segment.

## Case presentation

We evaluated three patients with ocular trauma and IOFBs in the anterior segment (summarized in Table 1). CT scanning was performed at the initial visit. Slit-lamp examination, UBM, and B-scan ultrasonography were further used for localization of the IOFBs. All procedures using B-scan ultrasonography were performed by the same technician, using the same ultrasonography instrument (model: B-Scan-Cinescan,Quantel Medical,France). All cataract extractions were performed under retrobulbar anesthesia by the same physician. Postoperative courses were all uneventful.

**Table 1:** Summary of three cases of IOFBs in the anterior segment

No	Case 1	Case 2	Case 3
Gender/Age	Male/32	Female/44	Male/43
Eye	Right	Left	Right
Time interval between injury and presentation	2 months	3 hours	5 days
Material of IOFB	Magnetic	Nonmagnetic	Magnetic
Clinical detection of IOFB	SL(−),CT(+), UBM(−),B(+)	SL(−),CT(+), UBM(−),B(+)	SL(−),CT(+), UBM(−),B(+)
IOFB entry route	Cornea	Cornea	Cornea
Status of the wound	Self-sealed	Small, sutured	Small, sutured
Traumatic cataract	Total	Total (lens cortex spillage)	Total
Posterior lens capsule status	Intact	Intact	Inferior rent (vitrectomy)
Post-operative RD	-	-	-
Initial BCVA	20/200	FC/50 cm	20/250
Final BCVA	20/20	20/25	20/40

*SL* slit-lamp exam, *CT* computed tomography, *UBM* ultrasound biomicroscopy; *B* B-scan ultrasonography, *FC* finger counting, *IOFB* intraocular foreign body, *RD* retinal detachment, *BCVA* best corrected visual acuity

### Case 1

**Fig. 1:**
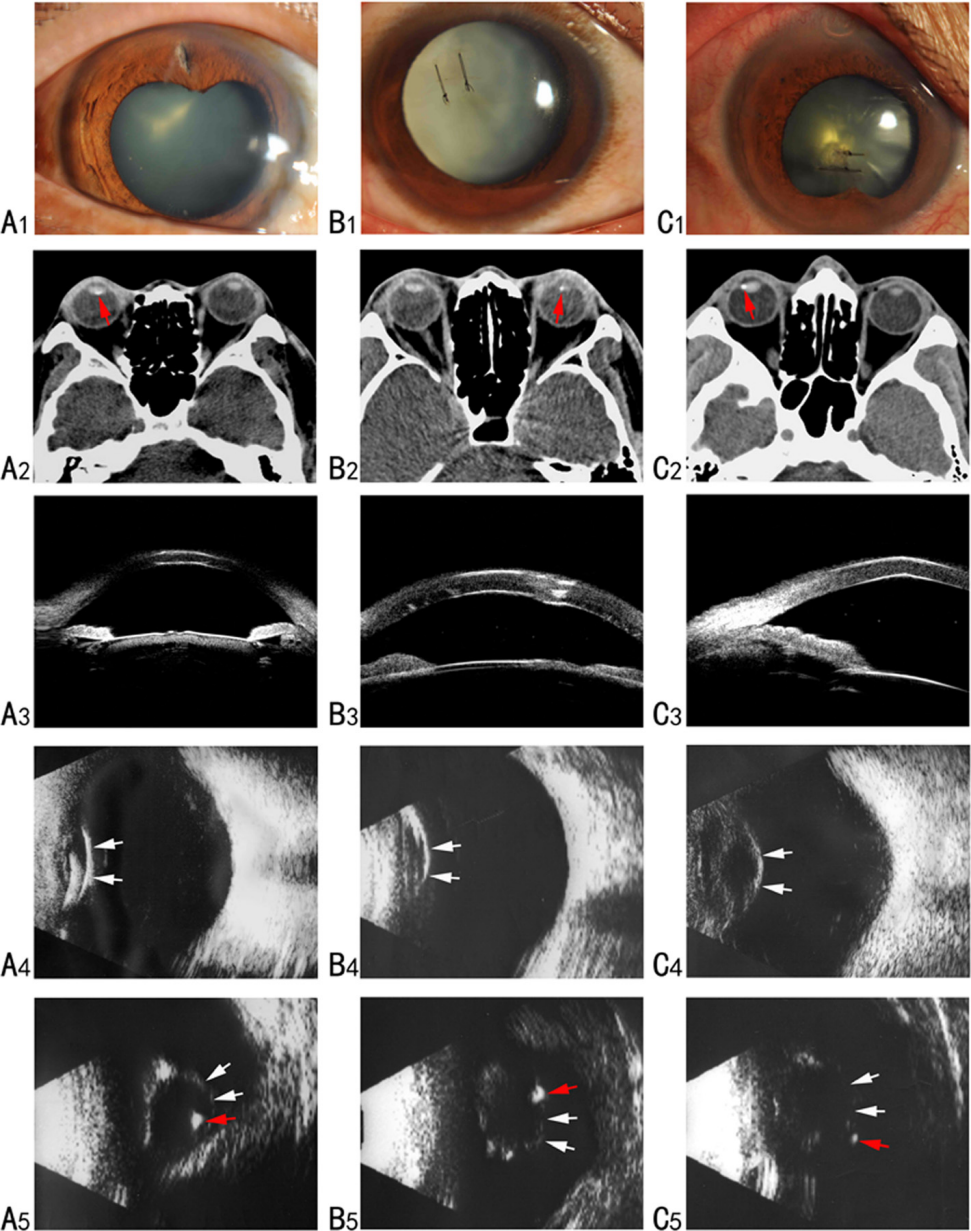
Preoperative photographs of the three patients. **a** Case 1, **b** case 2, **c** case 3. Slit-lamp photographs showing corneal laceration and lens opacity (**a**1-**c**1). CT scanning revealed intralenticular foreign bodies red arrow, **a**2-**c**2). UBM exam failed to detect any IOFB in the anterior segment (**a**3-**c**3). Axial scanning of B-scan ultrasonography shows the echo of the posterior surface of the lens (white arrow), with an abnormal echo in the posterior lens in case 1 (**a**4) but no evident IOFB in case 2 (**b**4) and case 3 (**c**4). Transverse scanning of B-scan ultrasonography clearly showed the position between the IOFB (red arrow) and the posterior lens capsule (white arrow)

A 32-year-old male patient presented with a history of injury to the right eye while grinding metal at work, 2 months prior to his initial visit. Upon examination, the patient’s best corrected visual acuity (BCVA) was 20/200 OD and 20/20 OS. The intraocular pressure (IOP) was normal in both eyes. A slit-lamp examination revealed a self-sealed and Seidel negative corneal perforation at the 12 o’clock position, an iris transillumination defect, and a lens opacity (Fig. 1a1). Fundus examination showed no abnormalities in both eyes. A CT scan showed an intralenticular foreign body in the right eye (Fig. 1a2), but UMB failed to detect any obvious IOFB in the anterior segment (Fig. 1a3). B-scan ultrasonography was further performed to identify the exact location of the IOFB and to assess the status of the posterior lens capsule. Axial scanning revealed a highly echogenic mass in the lens with a normal posterior segment (Fig. 1a4), whereas transverse scanning clearly localized the IOFB within the posterior lens, immediately in front of the posterior lens capsule (Fig. 1a5).

A surgical approach was planned to remove the IOFB and cataract, together with an intraocular lens implant. After capsulorhexis, cataract extraction was performed by phacoemulsification (Phaco). A viscoelastic agent was injected to allow the foreign body to be mobilized and removed by a Macpherson forceps through the 3.0-mm corneal section. A foldable posterior chamber intraocular lens (PC IOL) was placed in the capsular bag. Vitrectomy was not needed because of an intact posterior lens capsule. The postoperative course with topical corticosteroid treatment was uneventful. Two months after surgery, the patient’s corrected visual acuity recovered to 20/20.

### Case 2

A 44-year-old female patient presented in the emergency room with a 3-h history of pain and loss of vision, subsequent to an injury to the left eye by a stone. Her BCVA was FC/50 cm OS and 20/20 OD. The IOP was 18 mmHg in the left eye. Slit-lamp examination showed a 3-mm corneal laceration, a shallow anterior chamber, and lens opacity with ruptured anterior lens capsule (Fig. 1b1). CT scanning revealed an intralenticular foreign body in the left eye (Fig. 1b2). Fundus details were not visible because of the traumatic cataract. The corneal wound was repaired under retrobulbar anesthesia and the patient was sent to the ophthalmology clinic the following day. UMB examination was performed but failed to detect any IOFB in the anterior segment (Fig. 1b3). B-scan ultrasonography did not show any IOFB under axial scanning (Fig. 1b4), but transverse scanning clearly revealed that the IOFB was within the lens, just adjacent to the posterior lens capsule (Fig. 1b5).

Combined surgery with Phaco, PC IOL implantation, and IOFB removal were performed on the left eye. After capsulorhexis and cataract extraction with Phaco, a viscoelastic agent was injected and a nonmagnetic IOFB was successfully removed with forceps through the 3.0-mm corneal section, which was located at 10 o’clock, just adjacent to the posterior lens capsule. A foldable PC IOL was successfully implanted in the capsular bag and vitrectomy was avoided because of the intact posterior lens capsule. The postoperative course with topical corticosteroid treatment was uneventful. The patient’s BCVA recovered to 20/25 at 3 months after surgery.

### Case 3

A 43-year-old male patient complained of pain and loss of vision in the right eye for 3 h after being struck by a metal fragment at work 5 days prior to his initial visit. On presentation, his BCVA was 20/250 OD and 20/20 OS. IOP was normal in both eyes. Slit-lamp examination revealed a 2.5-mm penetrating cornea wound (sutured) and traumatic cataract (Fig. 1c1). CT scanning revealed an intralenticular foreign body (Fig. 1c2). The corneal wound had already been repaired under emergency surgery at a local hospital. Further examination was performed to localize the IOFB. A UBM examination failed to show any IOFB in the anterior segment (Fig. 1c3). B-scan ultrasonography did not show any IOFB under axial scanning (Fig. 1c4), while transverse scanning clearly localized the IOFB in the anterior vitreous just behind the posterior lens capsule (Fig. 1c5).

Phaco was performed along with vitrectomy. A magnetic IOFB was successfully removed and the PC IOL was implanted in the sulcus because of an inferior posterior capsular break. A retinal tear was found at the corresponding peripheral retina, and scleral cryotherapy was also performed. The postoperative period with topical corticosteroid treatment was uneventful. The patient’s visual acuity corrected to 20/40 at 1 month after surgery.

## Discussion

IOFBs following penetrating eye injury are common in most clinical cases, which can cause severe complications such as cataract, glaucoma, uveitis, retinal detachment, and endophthalmitis [6]. Intralenticular foreign bodies comprised only 5–10 % of all IOFBs [2, 3]. The treatment management and outcome depend on several factors such as size, location, material type, risk of infection, and other intraocular damage [7].

Because of the opacity of the cornea and traumatic cataract, it is usually difficult to get a direct visualization of IOFBs with ophthalmoscopy and slit-lamp biomicroscopy. Therefore, imaging is important in the evaluation of IOFBs. The present techniques available to detect IOFBs include CT scanning, magnetic resonance imaging (MRI), B-scan ultrasonography, UBM, and anterior segment optical coherence tomography (AS-OCT) [8]. CT scanning is considered the most sensitive imaging technique and can reveal IOFBs of various compositions. Orbital CT scanning has been performed with a 2- to 3.75-mm slice thickness [4], while the size of clinically encountered IOFBs have ranged from 0.5 to 25 mm [9]. Therefore, small and low-density IOFBs can be missed using CT scanning. In addition, artifacts of high-density IOFBs and amplification effects may influence the evaluation of intralenticular foreign bodies. MRI is time consuming and prone to motion artifacts. In addition, MRI is contraindicated in patients with magnetic IOFBs [10]. Both UBM and AS-OCT are well-established modalities for the diagnosis of lesions in the anterior segment [11]. UBM can identify IOFBs located in the posterior iris or ciliary body [5]. Unfortunately, with a 5.0-mm depth of penetration, it is difficult for UBM to detect IOFBs in the posterior lens [12]. In contrast, AS-OCT can visualize up to a depth of 6.0 mm, to determine the location and size of the intralenticular foreign body [13].

B-scan ultrasonography is usually used to detect posterior segment foreign bodies. The present report is the first to use B-scan ultrasonography to identify IOFBs in the anterior segment. Generally, penetrating wounds with IOFBs are common in work involving manual labor. County level hospitals in developing countries sometimes lack advanced equipment, such as UBM or AS-OCT, but almost every department of ophthalmology has a B-scan mode. Using transverse scanning along the nonaxial plane of the eyeball, B-scan ultrasonography can provide clear localization of IOFBs in the anterior segment, and can preoperatively evaluate the status of the posterior lens capsule. This information is very important for subsequent surgical planning. Although AS-OCT is a noncontact and noninvasive tool that has better resolution than B-scan ultrasonography, B-scan ultrasonography is a simpler and a more conventional method when dealing with cases with traumatic cataract and IOFBs involving the posterior region of the lens.

**Fig. 2:**
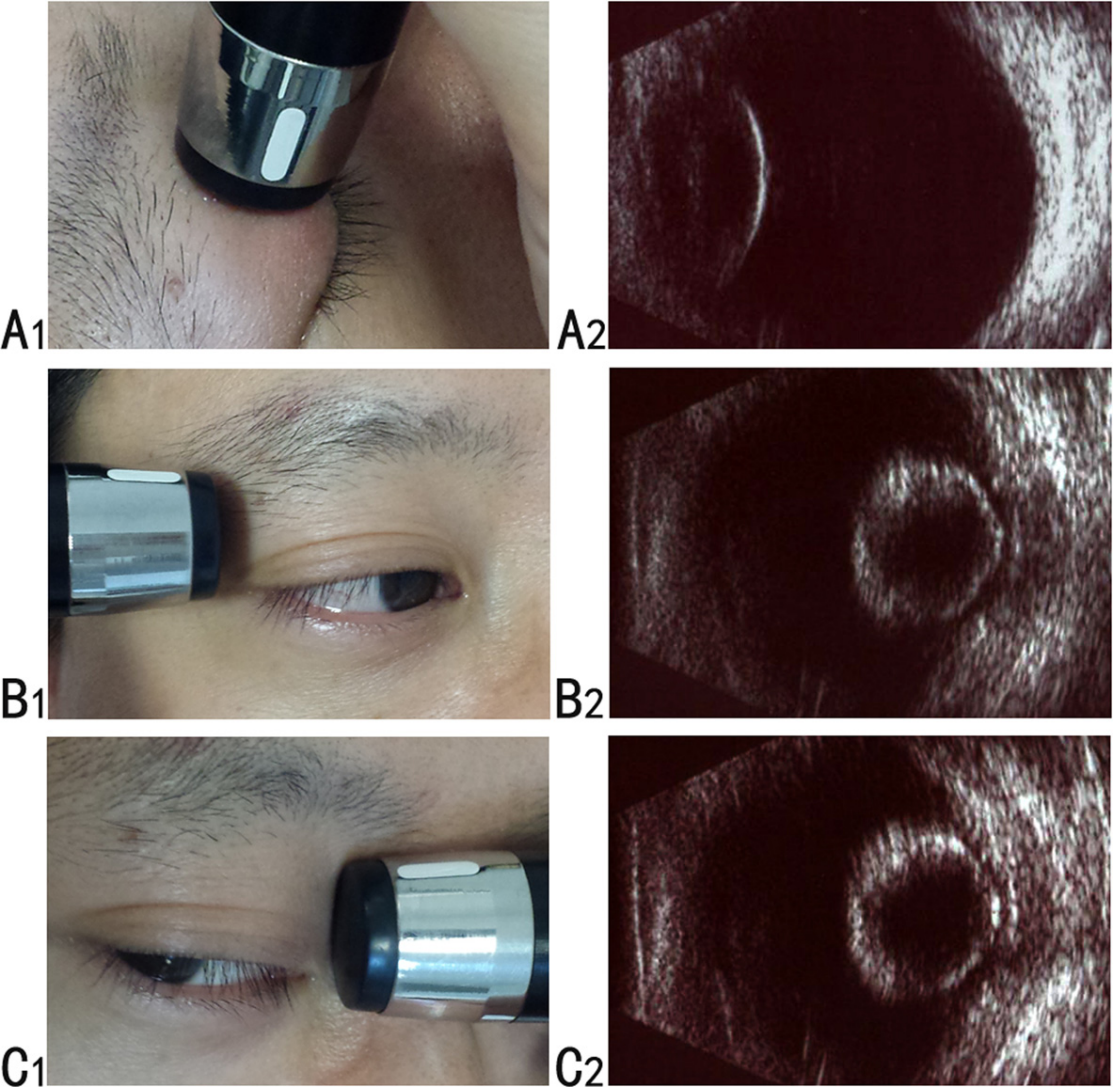
Schematics of operation procedures and corresponding images of B-scan ultrasonography. **a**1, commonly used axial scanning; **b**1, transverse scanning from the temporal side of the right eye with the eye turning to the left as far as possible; and **c**1, transverse scanning from the nasal side of the right eye with the eye turning to the right as far as possible. Only the echo of the posterior surface of the lens is shown under axial scanning (**a**2). Because of swelling of the lens after traumatic cataract, the echo of the whole lens can be displayed with transverse scanning (**b**2-**c**2)

There are several important issues regarding use of B-scan ultrasonography. First, B-scan ultrasonography is limited by its operator-dependent nature. A highly skilled ultrasonographer is very helpful when detecting the anterior segment. Routinely performed axial scanning (Fig. 2a1) can only display the echo of the posterior surface of the lens (Fig. 2a2) and always misses IOFBs near the lens. However, transversal scanning from the temporal (Fig. 2b1) and nasal side (Fig. 2c1), with the eye turning to the opposite position as far as possible, is able to show the entire lenticular echo, because of swelling of the lens in ocular trauma (Fig. 2b2-c2). Using this methodology, images of IOFBs and the posterior lens capsule can be clearly shown. Second, B-scan ultrasonography has the possibility of worsening globe damage with pressure from the ultrasound probe. Therefore, it should be only used after primary globe closure. Finally, case 1 and case 3 involved metallic IOFBs, but case 2 was a traumatic cataract with a nonmagnetic IOFB that involved a stone. Except for this case, we have had limited experience with other nonmetallic IOFBs, which are sometimes clinically found. When we encounter such cases in the future, we will apply B-scan technology to hopefully identify and localize other nonmetallic IOFBs.

## Conclusion

The accurate localization of IOFBs is very important for the optimum management of patients experiencing ocular trauma, especially when IOFBs are located in the anterior segment near the posterior lens capsule. Our cases demonstrated that B-scan ultrasonography was a simple and effective imaging modality for the localization of IOFBs in the anterior segment, and confirmed that transversal scanning was more suitable than axial scanning when detecting IOFBs in cases of traumatic cataracts.

## Consent

Written informed consent was obtained from the patients for publication of this case report and any accompanying images. A copy of the written consent is available for review by the Editor-in-Chief of this journal.

## Abbreviations

IOFBs: Intraocular foreign bodies
UBM: Ultrasound biomicroscopy
CT: Computed tomography
BCVA: Best corrected visual acuity
Phaco: Phacomulsification
PC IOL: posterior chamber intraocular lens
IOP: Intraocular pressure
MRI: Magnetic resonance imaging
AS-OCT: Anterior segment optical coherence tomography
